# Dual-platform affinity proteomics identifies links between the recurrence of ovarian carcinoma and proteins released into the tumor microenvironment

**DOI:** 10.7150/thno.37549

**Published:** 2019-08-22

**Authors:** Florian Finkernagel, Silke Reinartz, Maximiliane Schuldner, Alexandra Malz, Julia M. Jansen, Uwe Wagner, Thomas Worzfeld, Johannes Graumann, Elke Pogge von Strandmann, Rolf Müller

**Affiliations:** 1 Institute of Molecular Biology and Tumor Research (IMT), Center for Tumor Biology and Immunology (ZTI), Philipps University, Marburg, Germany; 2 Clinic for Gynecology, Gynecological Oncology and Gynecological Endocrinology, Center for Tumor Biology and Immunology (ZTI), Philipps University, Marburg, Germany; 3 Experimental Tumor Biology, Clinic for Hematology, Oncology and Immunology, Center for Tumor Biology and Immunology (ZTI), Philipps University, Marburg, Germany; 4 Clinic for Gynecology, Gynecological Oncology and Gynecological Endocrinology, University Hospital of Giessen and Marburg (UKGM), Marburg, Germany; 5 Institute of Pharmacology, Biochemical-Pharmacological Center (BPC), Philipps University, Marburg, Germany; 6 Department of Pharmacology, Max-Planck-Institute for Heart and Lung Research, Bad Nauheim, Germany; 7 Biomolecular Mass Spectrometry, Max-Planck-Institute for Heart and Lung Research, Bad Nauheim, Germany; 8 German Centre for Cardiovascular Research (DZHK), Partner Site Rhine-Main, Max-Planck-Institute for Heart and Lung Research, Bad Nauheim, Germany

## Abstract

The peritoneal fluid (ascites), replete with abundant tumor-promoting factors and extracellular vesicles (EVs) reflecting the tumor secretome, plays an essential role in ovarian high-grade serous carcinoma (HGSC) metastasis and immune suppression. A comprehensive picture of mediators impacting HGSC progression is, however, not available.

**Methods:** Proteins in ascites from HGSC patients were quantified by the aptamer-based SOMAscan affinity proteomic platform. SOMAscan data were analyzed by bioinformatic methods to reveal clinically relevant links and functional connections, and were validated using the antibody-based proximity extension assay (PEA) Olink platform. Mass spectrometry was used to identify proteins in extracellular microvesicles released by HGSC cells.

**Results:** Consistent with the clinical features of HGSC, 779 proteins in ascites identified by SOMAscan clustered into groups associated either with metastasis and a short relapse-free survival (RFS), or with immune regulation and a favorable RFS. In total, 346 proteins were linked to OC recurrence in either direction. Reanalysis of 214 of these proteins by PEA revealed an excellent median Spearman inter-platform correlation of ρ=0.82 for the 46 positively RFS-associated proteins in both datasets. Intriguingly, many proteins strongly associated with clinical outcome were constituents of extracellular vesicles. These include proteins either linked to a poor RFS, such as HSPA1A, BCAM and DKK1, or associated with a favorable outcome, such as the protein kinase LCK. Finally, based on these data we defined two protein signatures that clearly classify short-term and long-term relapse-free survivors.

**Conclusion:** The ascites secretome points to metastasis-promoting events and an anti-tumor response as the major determinants of the clinical outcome of HGSC. Relevant proteins include both bone fide secreted and vesicle-encapsulated polypeptides, many of which have previously not been linked to HGSC recurrence. Besides a deeper understanding of the HGSC microenvironment our data provide novel potential tools for HGSC patient stratification. Furthermore, the first large-scale inter-platform validation of SOMAscan and PEA will be invaluable for other studies using these affinity proteomics platforms.

## Introduction

Ovarian carcinoma is the most fatal of all gynecological malignancies and ranks fifth among all cancer-related deaths in women 1. Its most common and aggressive form is high-grade serous carcinoma (HGSC). Multiple features contribute to its fatal nature, one of which is the role of its distinct tumor microenvironment. This environment includes the peritoneal fluid, which mediates the metastatic spread within the peritoneal cavity. This occurs even at a very early stage of the disease when the tumor is still confined to its primary site, brought about by disruption of the outermost sheath lining the ovary or fallopian tube. At advanced stages tumor tissue is directly exposed to the peritoneal fluid (termed ascites when reaching larger volumes), and shed vast number of tumor and tumor-associated immune cells into this environment. The peritoneal fluid is rich in tumor-promoting soluble factors and extracellular vesicles (EVs) 2, creating a unique environment promoting tumor growth, progression, chemoresistance and immune evasion 2-6.

Several lines of evidence support the clinical significance of cytokines, growth factors, extracellular matrix (ECM) remodelers and other mediators in ascites. Evaluation of genomic data, for example, has identified a number of adverse clinical associations of signaling pathways established by polypeptide ligands and their receptors, including TGFβ, PDGF, VEGF, ephrins, CXCL12 and CCL chemokines and has pointed out a relevance for proteins involved in ECM remodeling 7, 8. Furthermore, several studies have demonstrated highly significant associations between the ascites levels of various cytokines measured by ELISA and relapse-free (RFS) or overall survival (OS) of ovarian cancer patients, for example TGFβ, IL-6, IL-10 and LIF 8-15. Comprehensive, systematic proteomic studies of the HGSC microenvironment have, however, not been performed. This is mainly due to the challenge posed by the massive dynamic range of blood-derived proteins, such as albumin and globulins, which limits the applicability of MS for the analysis of, for example, ascites 16-20. It is therefore likely that many clinically relevant mediators remain to be identified.

Although ovarian carcinoma is the deadliest cancer of the female reproductive tract with an average relapse-free survival after first-line therapy of less than two years, a small fraction of patients (<20%) remains relapse-free for more than 5 years, and a subset even considerably longer 21. To date, biomarkers are not available to reliably distinguish these patient populations. The only parameters strongly associated with a shorter time to relapse are the extent and success of the initial tumor debulking and primary platinum sensitivity, but they are of limited usefulness to reliably identify long-term survivors 22-25. The identification of prognostic markers would be of great clinical value - not least as a basis to adapt therapies to individual patients. To date, however, neither candidate protein marker approaches in tumor tissue or malignancy-associated ascites nor transcriptomic analyses have succeeded in defining clinically applicable markers or signatures 22, 26-28.

Recent achievements in proteomic technologies have improved the detection of biomarkers, but large-scale or unbiased analyses aiming at the prognosis of ovarian cancer have not been described. A particular interesting advance in this context are the recently developed competing affinity proteomics technology platforms SOMAscan 29 and proximity extension assay (PEA) 30, commercialized by SomaLogic and Olink, respectively. SOMAscan is based on the application of SOMAmers, aptamers or short, single-stranded DNA molecules selected for their ability to bind specific molecular targets. To achieve greater diversity and high affinity SOMAmers include non-natural nucleotides bearing functional groups mimicking amino acid side chains. In addition, SOMAmers are selected for low dissociation rates, enabling their use in quantification assays without the requirement for a secondary ligand. PEA, in contrast uses pairs of oligonucleotide-coupled antibodies binding epitopes in close proximity on the target protein. As a result, the covalently coupled oligonucleotides anneal into a target for proximity-dependent DNA polymerization, subsequently amplified by quantitative polymerase chain reaction (qPCR). A strong advantage of these affinity proteomic approaches over mass spectrometry (MS) is their applicability to unfractionated plasma and related fluids (including ascites) in spite of the high abundance of blood proteins. In view of the clinical relevance of cytokines, growth factors, ECM modifiers and other protein mediators in the HGSC environment, we applied these affinity proteomics platforms to the identification of clinically relevant and potentially prognostic biomarkers.

## Materials and Methods

### Plasma and ascites samples from patients

Peripheral blood and ascites were collected from patients with HGSC or benign conditions prior to surgery at Marburg University Hospital (Table S1). Peripheral blood was collected in lithium heparin collection tubes (16 I.E. heparin / ml blood) and diluted with an equal volume of PBS. Plasma was obtained by centrifugation. Cell-free ascites and plasma samples were cryo-preserved at -80^o^C. Samples were thawed for ELISA or preparation of shipment to SomaLogic or Olink on dry ice. The collection and the analysis of plasma and ascites samples was approved by the ethics committee at Philipps University (reference number 205/10). Donors provided written consent in accordance with the Declaration of Helsinki.

### Cells

Tumor cell spheroids, tumor-associated macrophages (TAMs) and tumor-associated T cells (TATs) were isolated from HGSC ascites as previously reported 8, 15. Tumor cell cultures from spheroids were established as published 31. Briefly, ascites-derived spheroids were cultured on a mixed-charged surface (Primaria culture dishes, Corning) in OCMI medium consisting of equal volumes of DMEM/Ham's F12 and M199 medium (Millipore) supplemented with 2 mM glutamine, 20µg/ml insulin, 10mM HEPES (pH 7.4), 10µg/ml transferrin, 0.2pg/ml triiodothyronine, 5µg/ml o-phosphoryl ethanolamine, 8ng/ml selenous acid, 25ng/ml all-trans retinoic acid, 500ng/ml hydrocortisone, 25ng/ml cholera toxin (all from Sigma Aldrich), 10ng/ml epidermal growth factor (Gibco), 5µg/ml linoleic acid (Cayman chemicals) and 5% FCS (Gibco). The HEK293 cell line (ACC-305, DSMZ) was cultured according to the DSMZ guidelines in DMEM.

### Isolation and characterization of EVs

EVs were collected from the supernatant of ovarian cancer cells cultivated in EV-depleted medium (overnight centrifugation at 100,000 x g) using sequential ultracentrifugation 32. The centrifugation protocol included consecutive pre-centrifugation steps at 300 x g (10 min), 2,000 x g (10 min) and 3,500 x g (20 min) for clearance of cells and cellular debris before ultracentrifugation at 10,000 x g (60 min) and/or 100,000 x g (90 min) using an SW41Ti or Type 45Ti rotor for at least twice with resuspension in PBS or HBSS. The amount of EV protein was quantified by Nanodrop 1000 (Thermo Scientific, Schwerte, Germany) and/or using a BCA assay (Pierce). The number of particles was determined by Nanoparticle Tracking Analysis (Particle Metrix).

### Western Blot

Cells or EVs were lysed in buffer (50 mM Tris-HCl pH 8, 150 mM NaCl, 0.5% Triton X-100, 0.5% protease inhibitors). Ten micrograms of total protein were analyzed by sodium dodecyl sulfate-polyacrylamide gel electrophoresis followed by blotting using standard methods.

### Proteomic analysis of EVs

EV samples were obtained by serial ultracentrifugation of ovarian cancer cell supernatants cultivated in vesicle depleted medium as described 32. EVs (corresponding to 10 µg protein) were lysed in 6M Urea / 2M thiourea in Hepes buffer additionally aided by by a -80°C freeze/thaw cycle. Proteins were then treated with 1:10 volume of 100 mM dithiothreitol for 30 minutes at RT and alkylated using 1:10 volume of 550 mM for 30 min and RT. Proteins were Lys-C digested for 2h at RT and afterwards solution was subsequently diluted to 1M urea using 50 mM ABC buffer for trypsin digestion overnight at RT (both digests at 1 to 100 enzyme to protein ratio). The reaction was stopped by acidification using 5% CAN / 0.2% trifluoroacetic acid before peptides were desalted on a C18 Stage tip.

All samples were analyzed at the CECAD proteomics facility (Cologne, Germany) on a Q-Exactive Plus (Thermo Scientific) mass spectrometer coupled to an EASY nLC 1000 UPLC (Thermo Scientific). Briefly, peptides were loaded with solvent A (0.1% formic acid in water) onto an in-house packed analytical column (50 cm × 75 µm I.D., filled with 2.7 µm Poroshell EC120 C18, Agilent). Peptides were chromatographically separated at a constant flow rate of 250 nl/min using linear gradient (solvent B 0.1% formic acid in 80% acetonitrile) over 240 min (total proteome) and 150 min (EVs) gradients. Mass spectrometric raw data were processed with MaxQuant (version 1.5.3.8) using default parameters. Data were deposited at PRIDE.

### Proteomic analysis of secretomes

Secretomes were determined by LC-MS/MS of conditioned medium from short-term cultures of primary cells isolated from HGSC ascites as described (Worzfeld et al., 2018).

### SOMAscan analysis of plasma and ascites samples

The SOMAscan platform has been described in detail before 29, 33-37. Briefly, citrate-plasma and citrate-ascites is diluted into three dilution bins (0.05, 1, 40%) and incubated with bin-specific collections of bead-coupled SOMAmers in a 96-well plate format. After washing, bead-bound proteins are biotinylated and complexes comprising biotinylated target proteins and fluorescence-labelled SOMAmers are photocleaved from the bead support and pooled. Following recapture on streptavidin beads and further washing steps, SOMAmers are eluted and quantified as a proxy to protein concentration by hybridization to arrays of SOMAmer-complementary oligonucleotides. Based on standard samples included on each plate, the resulting raw intensities are processed using a data analysis work flow including hybridization normalization, median signal normalization and signal calibration to control for inter-plate differences. One-hundred samples of ascites (n=70) and plasma (n=30) were analyzed by SomaLogic Inc. (Boulder Colorado, USA). Data for 1,305 SOMAmer probes (SOMAscan assay 1.3K) was obtained for these samples. Plasma samples were diluted 1:2 during preparation, which was accounted for by multiplying the measured SOMAscan units by 2. Since the biological relevance of very low concentrations of mediators in ascites is questionable and difficult to quantify, only SOMAmers yielding more than 1724 SOMAscan units (median of all samples in the dataset) in at least one sample were considered for further analyses.

### PEA (Olink) analysis of plasma and ascites samples

All 30 plasma samples and 20 of the ascites samples analyzed by SOMAscan were randomized in 96-well plates and covered with MicroAmp Clear Adhesive Film (Thermo Fisher Scientific) for PEA analysis at Olink 30, 38. To calculate intra- and inter-assay coefficients of variation (%CV), a pool of randomly selected plasma samples was used. All serum samples underwent one freeze-thaw cycle prior to proteomic analysis. Three hundred sixty eight markers in four 92-multiplex immunoassay panels (CVD II, Dev, Neuro I, Onc II) were measured simultaneously for each sample (details in Table S3 and on https://www.olink.com/products/complete-protein-biomarkers-list/). The Olink assay is based on the proximity extension assay (PEA) technology 30 using pairs of oligonucleotide-labeled antibodies as probes. These paired antibodies bind to the target protein in the sample in close proximity, allowing for the formation of a PCR target sequence by a proximity-dependent DNA polymerization, which is subsequently amplified by quantitative polymerase chain reaction (qPCR) using universal primers. Following the digestion of surplus primers quantification is performed using a microfluidic chip (96.96 Dynamic Array IFC, Fluidigm Biomark), run on a BioMark platform (BioMark HD System). For details see https://www.olink.com/data-you-can-trust/technology/.

### Analysis of ascites samples by ELISA

BCAM in ascites was quantified by ELISA (ELH-BCAM-2; BioCat GmbH, Heidelberg, Germany) according to the instructions of the manufacturer.

### Statistical analyses

Comparative data (Table 1) were statistically analyzed by unpaired Student's t-test (two-sided, equal variance) and adjusted for multiple hypothesis testing by Benjamini-Hochberg correction. Box pots depicting medians (line), upper and lower quartiles (box), range (whiskers) and outliers/fliers (diamonds) were constructed using the Seaborn boxplot function. Correlations were analyzed using the scipy.stat functions. Associations with relapse-free survival (logrank test), hazard ratio (HR) and median survival times were analyzed using the Python Lifelines KaplanMeierFitter and CoxPHFitter functions. All logrank test results are presented as nominal p-values.

### Definitions relevant to the development of signatures discriminating patients with long and short RFS

Short-term survivors are patients with relapsed cancer within 24 months after first-line surgery (uncensored RFS <24 months). Long-term survivors are patients with no relapse at 24 months (censored or uncensored RFS ≥24 months). Signatures score: the fraction of proteins in a combination of proteins (signature) above the best-fit threshold (quantile in Table 2) in individual patients. Type 1 signatures identify all long-term survivors, type 2 signatures all short-term survivors. For both signatures, a high score (score > length of signature / 2) predicts a short RFS, a low score (score < length of signature / 2) predicts a long RFS.

### Identification of signatures discriminating patients with long and short RFS

The 346 proteins negatively or positively associated with RFS (nominal p<0.05; Table S4) were included in an approach to identify prognostic signatures by random marker combinations. Since it is virtually impossible to test all possible combinations, we performed a step-wise analysis by first identifying a core signature with the most relevant 3 markers followed by gradually increasing the length of the signature.

These tests were carried out as follows: First, we determined whether the concentration of each protein in the ascites of each individual patient is below or above the best-fit threshold level (used for the determination of logrank p-values; see Table S4), and assigned of a score of 1 for all instances with a level below the threshold, otherwise a score of zero. Patient-wise addition of these scores and division by the number of proteins in the signature yielded the respective signature score. A signature score >0.5 was considered a signature fit for a given patient.

This approach identified the markers BCAM, HSPA1A and DKK1 as the core type 1 signature, which was able to correctly identify 90.9% all long-term survivors and 67.6% short-term survivors. Inclusion of Corticotropin-lipotropin and DPP7 yielded a type 1 signature that correctly identified 100% of long-term survivors and 76.5% of short-term survivors. Further inclusion of LMAN2 and CTSS increased the percentage of short-term survivors to 82.4 %. Further inclusion of for example RPSA and ARSB yielded the type 1 signature BCAM, HSPA1A, DKK1, Corticotropin-lipotropin, DPP7, LMAN2, CTSS, RPSA, ARSB, which correctly identified 100% of long-term survivors and 85.3 % of short-term survivors.

For type 2 signatures we identified the markers CAPG, LCK and TNFAIP6 as the core signature, which was able to correctly identify 91.2% of short-term survivors and 63.6% long-term survivors. Inclusion of REG1A yielded a type 2 signature that correctly identified 100% of short-term survivors and 59.1% of long-term survivors. Further inclusion of CTSZ, ARSA and RPS7 increased the percentage of long-term survivors to 81.8%. Further inclusion of for example CD27 and TNFAIP6 yielded the type 2 signature CAPG, LCK, TNFAIP6, REG1A, CTSZ, ARSA, RPS7, CD27, CRLF1, which correctly identified 100% of short-term survivors and 86.4% long-term survivors.

Next, we tested all combinations of the best type 1 and 2 signatures for optimal performance, defined as the percentage of correctly identified short-term and long-term survivors. To this end, we identified those patients, for which both signatures were consistent (i.e., above or below the threshold; (filled circles in Fig. 8A). Inconsistent instances were considered as "prediction not possible" (asterisks in Fig. 8A). For consistent instances, predictions were considered either "short RFS" for combined signature scores (added signature 1 and 2 scores) above 50% of the maximally possible score, or “long RFS” for combined signature scores below 50% of the maximally possible score. The maximally possible combined signature score is the sum of the lengths of both signatures.

## Results

### SOMAscan-based proteomics of OC ascites

In the first part of this study, we sought to identify biomarkers with potential clinical relevance in the ascites fluid of OC patients. Toward this goal, we determined the relative concentration of 1,305 plasma proteins in HGSC ascites (n=70 samples; Table S1) and compared these to corresponding values obtained with plasma either from HGSOC patients (OC-plasma, n=20; Table S1) or plasma from patients with non-malignant diseases (N-plasma, n=10; Table S1) using the SOMAscan technology (Table S2). In this context it must be stressed that affinity proteomic approaches represent “epitopomic” rather than proteomic data, and changes in signal may derive from protein abundance as well as epitope occlusion through post translational modification, single nucleotide polymorphisms etc..

**Figure 1:**
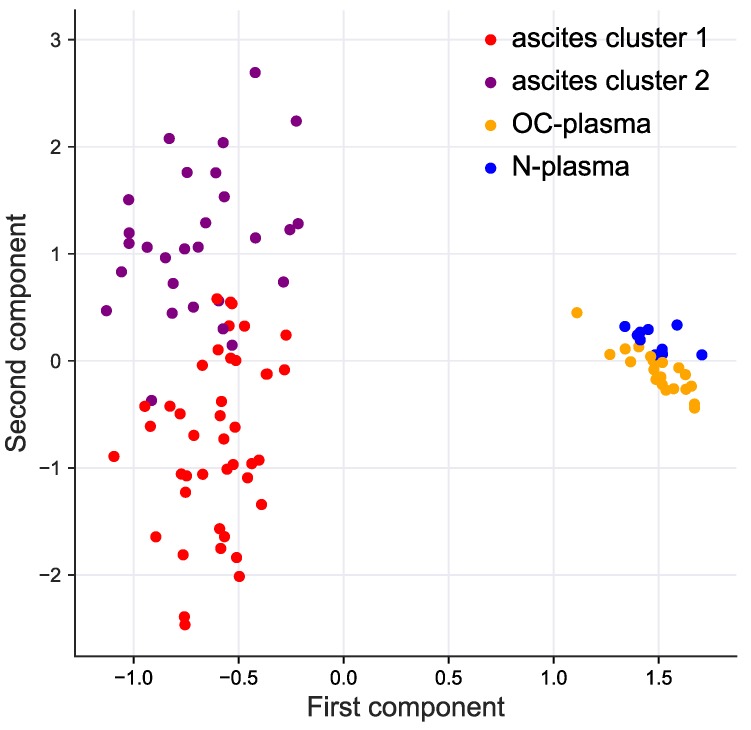
** Clustering of ascites and plasma samples based on SOMAscan signals.** The plot shows the results of a principal component analysis (PCA) of ascites samples (purple and red), HGSC-plasma samples (orange) and plasma samples from patients with non-malignant diseases (blue) samples.

Both, hierarchical clustering (Fig. S1) and principal component analysis (PCA; Fig. 1) achieved a clear separation of ascites and plasma samples. Consistent with this observation, we defined 356 proteins with significantly higher signal levels in ascites versus plasma (p-value <0.05 by two-sided unpaired t-test and adjusted for multiple hypothesis testing by Benjamini-Hochberg correction; Table S3). Of these, 45 were at least 5-fold elevated in ascites relative to plasma (Table 1).

These strongly upregulated protein signals include several factors with a previously reported role in promoting OC progression and/or an association with poor clinical outcome such as IL-6 13, 14, 39, TIMP1 40, KLK11 41, 42, VEGF123/VEGF-A 43, 44 and osteonectin/SPOCK2 45. However, studying proteins with increased SOMAscan signal also identified numerous other factors previously not described in OC ascites. Included intracellular proteins as heat shock proteins and protein kinases are presumably released from cells through non-canonical pathways, such as EVs.

**Figure 2:**
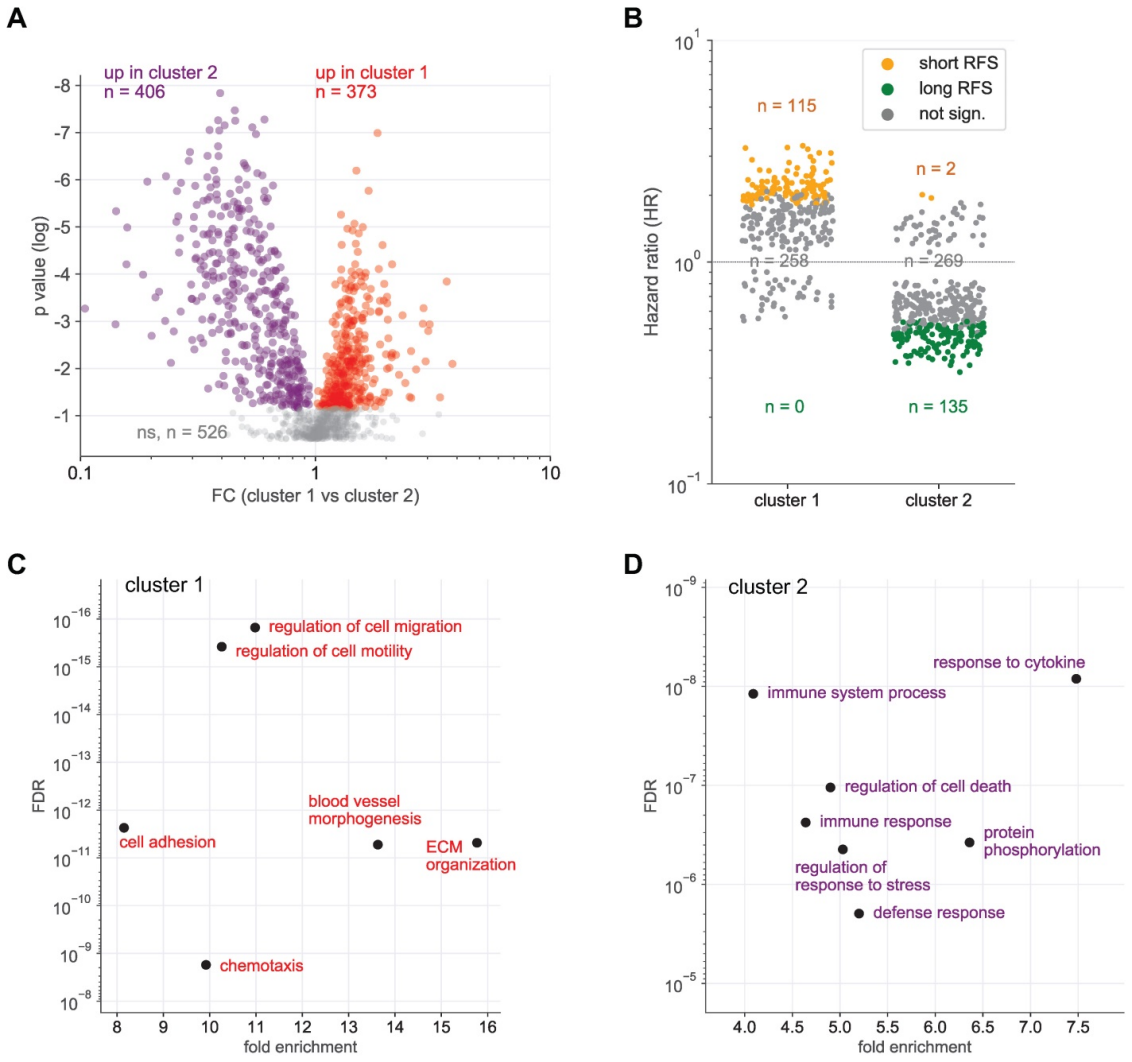
** Identification of SOMAscan protein signals differentially regulated in ascites clusters 1 and 2. (A)** Volcano plot showing the protein signals significantly upregulated in cluster 1 in red and in cluster 2 in blue. **(B)** Opposite linkage of cluster 1 and 2 protein signals in ascites to relapse-free survival (RFS). The plot shows the hazard ratios (HR) for cluster 1 (red) and cluster 2 proteins (blue). Long RFS: logrank p <0.05 (for RFS) and HR<1. Short RFS: logrank p <0.05 and HR>1; not sign.: logrank p ≥0.05.** (C)** Functional annotation of proteins underlying the upregulated signal in cluster 1 by gene ontology (GO) enrichment analysis. p values are plotted against fold enrichment. Only specific non-redundant terms with p values <10^-8^ and fold enrichment ≥8 are shown. **(D)** Functional annotation of proteins underlying the upregulated signal in cluster 2 analogous to panel C.

Hierarchical clustering further identified 2 clearly separable clusters in ascites (ascites cluster 1 and 2 in Fig. 1 and S1). The signals of 373 proteins were significantly higher in cluster 1 (versus cluster 2), while 406 proteins showed an opposite pattern (Fig. 2A). Intriguingly, the majority of proteins in cluster 1 are associated with a short RFS of ovarian cancer (n=115 versus 0 linked to a long RFS; Fig. 2B). By contrast, most proteins in cluster 2 are associated with a favorable RFS (n=135 versus 2 linked to a short RFS; Fig. 2B). PANTHER gene ontology (GO) enrichment analysis 46 (http://www.geneontology.org) revealed that cluster 1 is predominantly associated with metastasis-associated biological processes, such as cell motility, migration, adhesion, extracellular matrix remodeling (ECM) and angiogenesis (Fig. 2C). This is in agreement with the observed association of these proteins with a short relapse-free survival (RFS) (Fig. 2B). On the contrary, cluster 2 is mainly linked to immune regulation (Fig. 2D). Taken together with the RFS data in Fig. 2B, this suggests that these proteins play a role in the anti-tumor immune defense. The observations are further consistent with the biological features known to determine the outcome of ovarian cancer, suggesting that proteins in ascites may impact progression of the disease and be of prognostic relevance.

### Associations of individual proteins in ascites with relapse-free survival

To identify biomarkers with a potentially prognostic value we determined the association of all protein signals in ascites measured by SOMAscan with the RFS of the patients. A nominal logrank p-value <0.05 was observed for 346 of the signals (Table 2 and Table S4). These include (i) bona fide secreted proteins (such as cytokines, growth factors, hormones, proteases and extracellular matrix components), (ii) membrane receptors (e.g., BMPR2, EPHA5, EPHB2, EPH6, ERBB1, LEPR, IL1R1, IL2RG, IL13R1, IL15RA, IL17RC, IL18R1, IL22RA1, IL27RA1, PGGFRA) that may be released as soluble forms, produced by proteolytic cleavage or present on EVs, and (iii) intracellular proteins (for example protein kinases) which might also be constituents of EVs or released from necrotic cells. A number of these intracellular proteins represent cell-type-specific components (such as LCK) and may thus be useful as surrogate markers for immune cells in the tumor environment, including ascites.

The data show both adverse and favorable associations with disease outcome, i.e., protein signals associated with either a positive hazard ratio (HR) or a negative HR. These comprise proteins with reported links to a short survival (Table 2), e.g., IL6, TGFB and VEGF 13, 15, 47, but also numerous proteins with hitherto unknown associations to OC survival in either direction.

**Figure 3:**
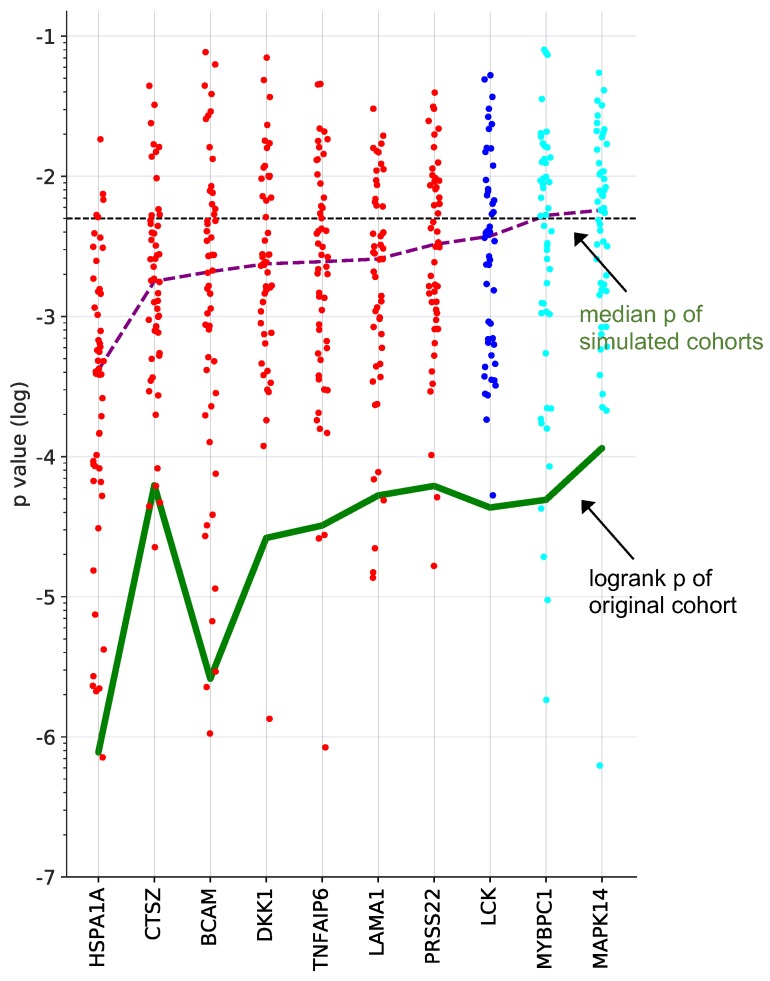
** Survival associations of SOMAscan protein signals in ascites.** Performance of the top 10 RFS-associated protein signals in simulated training and validation cohorts. Samples were randomly divided into two equally sized groups (simulated cohorts) and logrank p-values were determined for both cohorts. Dot plots illustrating the distribution of p-values for 25 simulations, i.e., 50 simulated cohorts, ordered by resulting median logrank-p values (purple line). The green line indicates the logrank p-values of the original dataset. Red: at least 50% of simulated datasets yielded significant p-values for both cohorts and a positive HR. Blue: at least 50% of the simulated yielded significant p-values for both cohorts and a negative HR. Cyan: less than 50% of the simulated datasets yielded significant p-values for both cohorts and a negative HR. The dashed line indicates the p=0.05 threshold.

**Figure 4:**
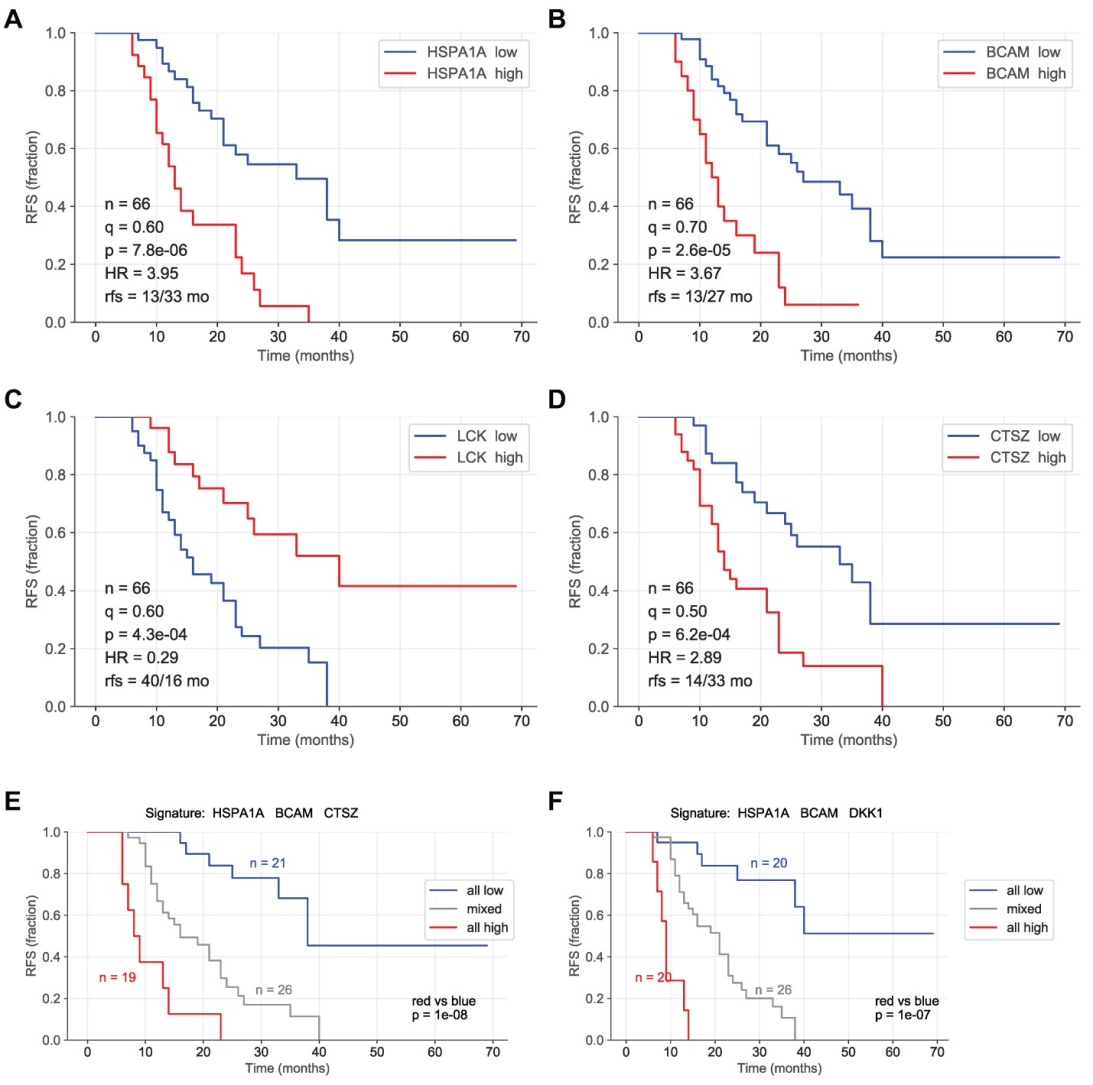
** Kaplan-Meier plots showing RFS associations of SOMAscan protein signals in HGSC ascites. (A-D)** Relationship between RFS and protein signals in ascites for HSP1A1, BCAM, LCK and CTSZ. n: number of evaluable patients. q: best-fit quantile; p: logrank p-value; HR: hazard ration; rfs: median RFS (months) in samples with high signal levels versus samples with low levels (dichotomized at the indicated best-fit quantile). **(E)** Patients were trichotomized for RFS analysis, using the best fit thresholds determined in panels A, B and D: Red: HSP1A1, BCAM, and CTSZ high; blue 2: HSP1A1, BCAM and CTSZ low; group 3: mixed high and low. See Materials and Methods for details.

To identify proteins with a robust association with RFS, we performed simulation studies with the 10 proteins yielding the lowest logrank p values (green line in Fig. 3). To this end, we split the patient cohort randomly into two sets of equal size (simulated cohorts; n=33 each) 25-times and determined the logrank p values for each of the 50 simulated cohort and each of the ten protein markers (dots in Fig. 3). Nine of these proteins yielded a median p value <0.05 (dashed line in Fig. 3), indicating robust associations with short (red) or long (blue) RFS. Examples of Kaplan-Maier plots for both positively and negatively associated protein signals are shown in Fig. 4A-D. HSPA1A (heat shock protein 70) showed an exceptionally high significance (nominal p=7.8x10^-6^; Figs. 3, 4A) and robustness in the simulation setting (Fig. 3), followed by BCAM (basal cell adhesion molecule; p=2.6x10^-5^; Figs. 3, 4B), CTSZ (cathepsin Z; p=6.2x10^-4^; Figs. 3, 4D) and DKK1 (Dickkopf-1; p=2.6x10^-4^; Fig. 3).

A particularly striking association was observed when patients were compared with high HSPA1A, BCAM and CTSZ to those with low signals of these proteins (Fig. 4E; p=10^-8^). A very similar finding was made for a combination of HSPA1A, BCAM and CTSZ (Fig. 4E; p=10^-7^). Any other combination of 2-5 proteins was considerably less significant (p<10^-4^; data not shown).

### Correlation of SOMAscan and Olink data

To assess the validity of the SOMAscan results we reanalyzed all 30 plasma and 20 ascites samples by means of the antibody-based Olink affinity proteomics platform. Of the disease centered 92-multiplex panels offered by Olink, we choose those with the greatest combined overlap to the SOMAscan 1.3k panel, i.e., CVD II, Dev, Neuro I, Onc II. As a result, 214 of the probed proteins were also contained in our SOMAscan analysis (Table S5), of which 48 were significantly associated with the RFS of HGSC patients based on SOMAscan data (Table 2 and Table S4). Spearman analysis across the 50 samples analyzed revealed a remarkable positive median correlation of ρ=0.73 for all 214 markers (Fig. 4A; Table S5) and even stronger median correlation of ρ=0.82 for the RFS-associated markers with only 8/48 markers with ρ<0.5 (Fig. 4B). Importantly, the SOMAscan measurements for several markers identified as strongly associated with poor RFS were validated by the Olink assay, including BCAM and CTSZ (ρ values of 0.95 and 0.87, respectively; Fig. 4B and C; Fig. S2). We also determined BCAM levels in ascites samples by ELISA, which further confirmed the SOMAscan data (ρ=0.92).

### Origin of RFS-associated proteins in ascites

To elucidate the potential cellular origin of the RFS-associated proteins we made use of our previous analysis of the secretomes of tumor cells, TAMs and TATs from HGSC ascites in short-term culture 8, 48. Intersecting the markers in the signatures established above with that data set (Fig. 6) revealed several comprised proteins as selectively secreted by one cell type, i.e., BCAM, GSTP1 and HSPA1A by tumor cells, CTSZ and TNFAIP6 by TAMs and LCK by TATs, while others originate from all cell types at similar levels (e.g., LAMA1, MMP16 or TAGLN2).

A large fraction of the RFS-associated proteins are intracellular or membrane proteins that are not secreted by canonical mechanisms. To shed light on the possible origin of these proteins, we analyzed potential correlations of extracellular vesicle (EV) markers with highly abundant RFS-associated protein signals (>10.000 SOMAscan units) in ascites 49-51. The heatmap in Fig. 7A shows that the concentration of several of these proteins correlates with the level of EV markers, including HSPA1A, BCAM, GSTP1, CAPG and TAGLN2, suggesting that EV-encapsulated proteins contribute to clinically relevant components of the HGSC secretome.

To test this prediction we determined the proteome of EVs isolated from the supernatants of tumor cell cultures from 3 different HGSC patients by MS. All three cell lines produced EVs with slight increase in size compared to HEK293 control cells (mean diameter 150 nm versus 130 nm as measured by Nanosight tracking analysis, Particle Metrix), but EV production per cell increased by in excess of 20-fold (Fig. 7B). Proteome analysis by MS identified 2162 proteins in the tumor-cell-derived EVs, which in the vast majority of cases were present at very similar levels in the 3 samples (Table S6). Of these, 318 were also found in ascites by SOMAscan (Table S2), including the top RFS-associated proteins HSPA1A, BCAM and DKK1 (Fig. 7C). The presence of HSPA1A in EVs was further confirmed by Western blotting (Fig. 7D). Taken together, these data strongly suggest that protein constituents of EVs play a major role in determining the survival-associated secretome of the HGSC microenvironment.

### Identification of potential biomarker signatures

Finally, we pursued an unbiased multivariate approach to assess the possibility to develop prognostic signatures based on the proteomic profiles of ascites. Toward this goal, we tested combinations of proteins that are directly or inversely associated with RFS with nominal significance (Table 2) for their power to discriminate short-term and long-term relapse-free survivors.

As described in detail in Methods and Methods, we were able to build signatures which identified either all long-term relapse-free survivors (type 1 signatures) or all short-term relapse-free survivors (type 2 signatures). However, both signatures types also yielded false positives.

We identified the markers BCAM, HSPA1A and DKK1 as the core type 1 signature, which correctly identified 90.9% all long-term survivors and 67.6% of all short-term survivors. Extension of this core signature by additional marker signals considerably improved its performance up to a length of 9 proteins. Thus, the combination of BCAM, HSPA1A, DKK1, Corticotropin-lipotropin (POMC), DPP7, LMAN2, CTSS, RPSA, ARSB correctly identified 100% of long-term survivors and 85.3 % of short-term survivors.

The markers CAPG, LCK and TNFAIP6 were identified as the core type 2 signature, which correctly identified 91.2% of short-term survivors and 63.6% long-term survivors. Addition of further marker signals up to a total size of 9 proteins incrementally improved performance of the signature: the combination of CAPG, LCK, TNFAIP6, REG1A, CTSZ, ARSA, RPS7, CD27, CRLF1 correctly identified 100% of short-term survivors and 86.4% long-term survivors.

Since none of these signatures reliably discriminated both groups of patients, we tested combinations of type 1 and 2 signatures for best performance, defined as the percentage of correctly identified short-term and long-term relapse-free survivors. 1464 combinations of type 1 and type 2 signatures correctly identified the clinical outcome (RFS) for 77% of all patients with no false predictions (Table S7). The result for one of these combinations of signatures is illustrated in detail in Fig. 8A. The robustness of this prediction was confirmed by the bootstrapping analysis in Fig. 8B, which yielded a median of 77% (with the 95% confidence interval ranging from 66 to 88%) for the correct detection of short-term and long-term survivors with no false predictions.

## Discussion

In the present study, we have analyzed 1305 plasma proteins in the ascites from HGSC patients using the aptamer-based SOMAscan technology. Hierarchical clustering of ascites samples identified two clusters. While the vast majority of protein signals upregulated in cluster 1 were associated with a short RFS and metastasis-linked biological processes, most protein signals upregulated in cluster 2 were associated with a favorable RFS and immune functions (Fig. 2). This is consistent with the biological features relevant to the outcome of HGSC, i.e., peritoneal adhesion and invasion by cancer cells and a T-cell-mediated cytotoxic response. These findings therefore suggest that the protein signals in ascites measured by SOMAscan parallel the biology and outcome of the disease in individual patients and may thus provide prognostic tools to assess the expected clinical course.

### RFS-associated proteins as cargo of EVs in ascites

Our data indicate that EVs play a major role in shaping the RFS-associated secretome of the tumor microenvironment (Fig. 7). This is consistent with previous proteomic studies of EVs from prostate and bladder cancer cell lines 52, 53 or blood plasma 35, which contained many of the intracellular and membrane proteins we detected in HGSC ascites. An important technical implication of this finding is that the conditions of any diagnostic, prognostic or predictive assay should allow for the detection of such proteins, for example, by including EV-disrupting detergents. The standard conditions under which the SOMAscan array operates with plasma or serum include 0.05% Tween-20 53 which presumably allowed for the detection of EV-associated proteins in our ascites samples. However, higher detergent concentrations may further improve the detectability of such proteins 53.

### Proteins in ascites associated with clinical outcome

Alignment of SOMAscan and clinical data led to the identification of 346 protein signals linked to RFS with nominal significance by logrank test (Table 2). Proteins associated with a short RFS (HR>1) include a number of cytokines and growth factors already associated with a poor survival in previous studies, for example CCL18, CXCL16, CTGF, several ephrin family members, IL6, HGF, TGFB1 2, 13, 15, 54-59 the secreted inhibitor of β‐catenin‐dependent Wnt signaling DKK1 60, 61 as well as the extracellular matrix protein LAMA1 8. However, numerous RFS-associated proteins identified in the present study have to date not been discussed in the context of the OC microenvironment. These include proteins with the strongest and most stable association with a poor clinical outcome, i.e., HSPA1A, BCAM, CTSZ and DKK1 (Fig. 3), and are therefore discussed in more detail below.

### Cross-validation of SOMAscan and PEA data

SOMAscan and PEA represent two affinity proteomics solutions. While these assays share the principal characteristics of measuring protein signals through non-covalently interacting binders and detection/quantitation by proxy through nucleic acids, they differ in the molecular nature of the binders employed (modified aptamers vs. natural antibodies), as well as detection technology (hybridization to a chip carrying aptamer-complementary nucleotides vs. qPCR) and commercialization strategy, which for SOMAscan targets screens of the whole probe set available, while Olink offers disease/organ centric subpanels with overlapping protein targets. An additional central difference is the reliance of PEA on two specific probes, which may increase confidence in *positive* PEA signals as compared to SOMAscan data.

After screening for protein signals associated with RFS in OC using the 1.3k version of SOMAscan, we sought to replicate the findings using an independent technology platform and used the four Olink-offered PEA panels providing the biggest overlap with the factors with the strongest RFS association (panels CVD II, Dev, Neuro I, Onc II; 214 unique proteins probed, 48 RFS-associated according to SOMAscan screen). This provided the opportunity for a limited scope cross-validation of the SOMAscan and PEA platforms. The strong median correlation between the data delivered by the two approaches not only largely validates our findings, but also strongly suggests a bulk equivalency of the tool kits. Under the assumption that the (compound) epitopes targeted are different for the assays, strong correlation may also be cautiously interpreted as corroborating evidence for a true protein abundance difference rather than an epitope effect (through e.g. occlusion by SNP or posttranslational modification). As e.g. massive differences in global protein glycosylation may still produce differential assay signals in the context of maintained protein abundance, such conclusions must be considered with caution. In cases where the correlation between the assays is negative, assay-differential epitope effects as well as lack of probe specificity may explain the apparent contradictory results.

### HSPA1A, BCAM and CTSZ as indicators of a short RFS

HSPA1A (HSP70), like other heat shock proteins, not only functions as an intracellular chaperone, but is also released into the extracellular space where it interacts with multiple surface receptors to modulate the function of other cells 62. It is not secreted by the classical signal-peptide pathway, but is released by exocytotic mechanisms, notably EVs 63, 64 which is in agreement with our finding of HSPA1A-comprising EVs in HGSC ascites and the supernatants from cultured patient-derived HGSC cells (Fig. 7). The extracellular functions of HSPA1A in the context of tumorigenesis are mediated by numerous cytotoxicity, scavenger and signaling receptors, but details remain contentious 65, 66. Accordingly, extracellular HSPA1A is thought to have immune modulatory functions, for instance in facilitating the cross-presentation of immunogenic peptides by major histocompatibility complex (MHC) antigens as well as in stimulating innate immune responses, but it has also been linked to therapy resistance, metastasis and poor clinical outcome in different cancer entities 62. Consistent with these variate activities of HSP70 it was reported that membrane-bound and extracellular HSP70 derived from tumor cells may induce effective anti-tumor immune responses 67. In the present study, the HSPA1A signals and thus likely the protein level in ascites showed by far the strongest association with a poor clinical outcome among all 1305 proteins analyzed (Fig. 3A and 4). It will therefore be of great interest to unravel the molecular and cellular mechanisms through which HSPA1A-bearing EVs impinge on metastasis-associated processes and immune cell functions in the HGSC microenvironment.

BCAM, also referred to as Lutheran antigen or CD239, is a cell adhesion molecule acting as a laminin receptor 68, and has been reported to promote cell migration in several models 69-71. Of particular interest in the context of our findings may be the observation that BCAM and laminin-55 have been reported to mediate the interaction of tumor cells and the endothelium to promote the metastatic spreading of colon cancer cells 72. As BCAM is a constituents of EVs released by HGSC cells (Fig. 7) it is tantalizing to speculate that EVs may act as a intercellular bridge facilitating the interaction of cancer cells with mesothelial and/or endothelial cells as part of the metastatic process in OC. It is consistent with our findings that OC is the cancer entity with the highest level of BCAM RNA and protein expression in the Human Protein Atlas (https://www.proteinatlas.org/ENSG00000187244-BCAM/pathology). Taken together, our data provide strong evidence for a clinically relevant role for BCAM in HGSC progression, presumably by promoting its metastatic spread.

CTSZ is a member of the cathepsin family of lysosomal cysteine proteinases exhibiting carboxy-peptidase activity 73. It is also secreted into the extracellular space 74, which presumably accounts for its presence in ascites, as, in contrast to HSPA1A and BCAM, CTSZ does not appear to be associated with EVs (Fig. 7A, Table S6). Like other cathepsins, CTSZ has been shown to promote metastasis in different models and cancer entities 73, 75, 76 for example by inducing epithelial-mesenchymal transition in hepatocellular carcinoma 77. In pancreatic neuroendocrine tumors, tumor-promoting functions of CTSZ were not dependent on its catalytic activity but instead were mediated via the Arg-Gly-Asp (RGD) motif in the enzyme prodomain, which regulated interactions with the extracellular matrix 75. Several cathepsins have been linked to metastasis and/or clinical outcome of stromal OC 74, 77-80, but CTSZ specifically has not been investigated in the context of OC to date. Interestingly, TAMs appear to be a major source of CTSZ in HGSC ascites, consistent with the crucial role of macrophage-derived CTSZ in pancreatic neuroendocrine tumors 75.

### Proteins in ascites associated with a favorable clinical outcome

We also identified a number of proteins in ascites that are strongly associated with a longer RFS (Fig. 4; Table 2). These fall into two functional groups, i.e. cytokines and intracellular protein kinases, in particular LCK, MAPK14 (p38), STK17B (DRAK2), CAMK2B and CAMK2D. The cytokines of the first group include L1A, IL36A and CCL13, which, as immune stimulatory mediators, may contribute to an anti-tumor immune response and thus a favorable clinical outcome. As these cytokines have, however, not been analyzed in the context of OC, their potential tumor suppressive functions remain obscure. It is likely that the protein kinases of the second group are constituents of EVs, even though none of these proteins were detected in EVs isolated from HGSC cultures (Table S6). The latter suggests that these protein kinases may be part of the cargo of EVs released by tumor-associated host cells or are derived from apoptotic or decaying cells. That the signal from these proteins in ascites does not correlate with the concentration of cytochrome c (CYCS) or lactate dehydrogenase (LDHA, LDHB; Table S2), however, renders the latter hypothesis unlikely. If these protein kinases are indeed constituents of EVs, their effect should be tumor suppressive. For at least two of these kinases tumor suppressive functions have been described, i.e., MAPK14 81 and STK17B 82. Elucidation of the potential molecular and cellular functions of these protein kinases in controlling HGSC progression will be an intriguing subject of future studies.

### Prognostic biomarkers and signatures

**Figure 5:**
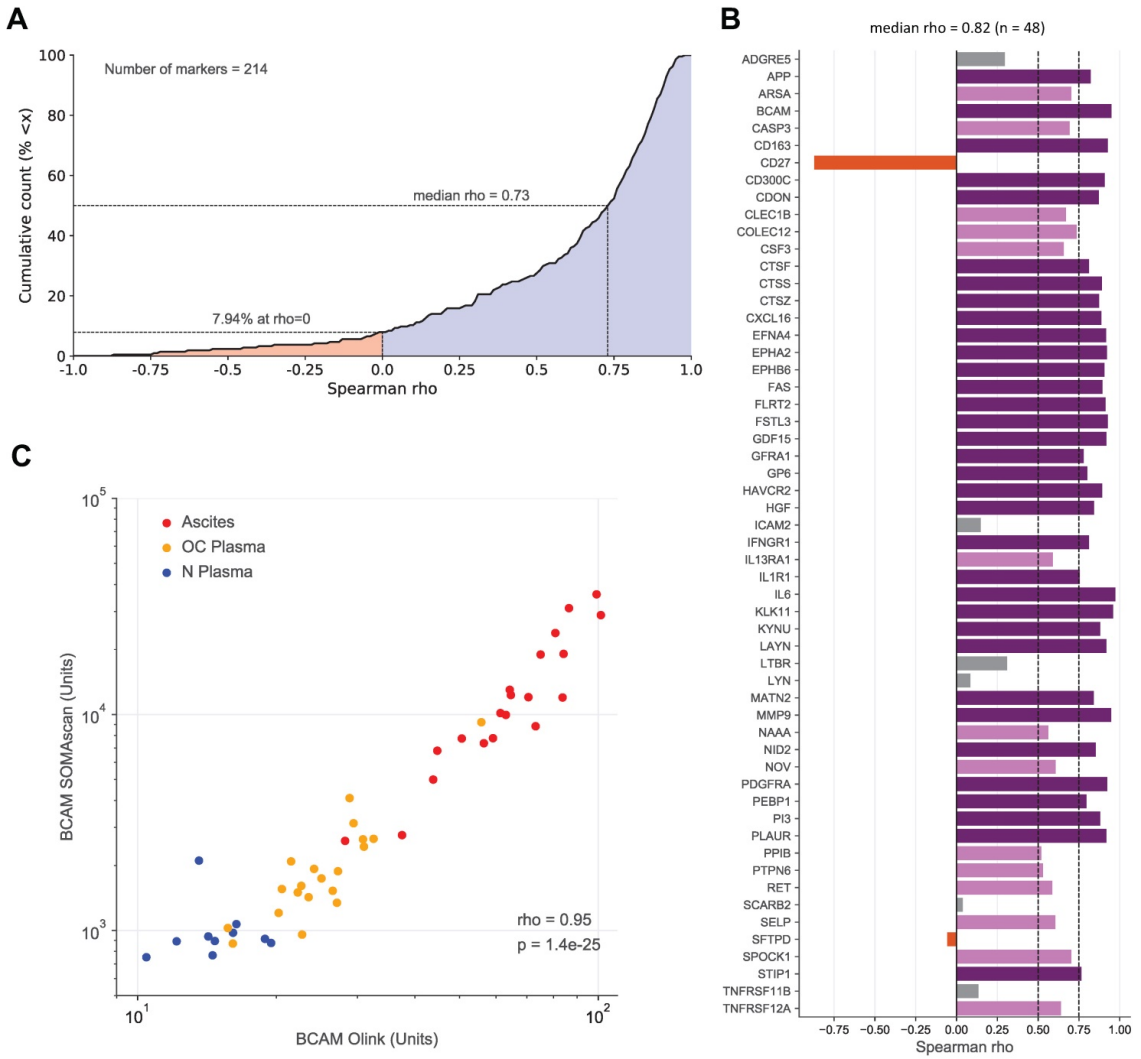
** Correlation of SOMAscan and PEA data for markers in ascites and plasma. (A)** Data for 214 markers in 10 N-plasma, 20 OC-plasma and 20 ascites samples were analyzed to calculate the cumulative distribution of Spearman correlation coefficients (ρ) between SOMAscan and PEA signal intensities, resulting in a median value of ρ = 0.73. The light blue area indicates positive correlations (92.52% of all instances), light red indicates negative correlations (7.48%).** (B)** Spearman correlations for all markers associated with RFS (SOMAscan) and present in the Olink panel measured (n=48) in the same datasets as in (A). Dark purple: ρ>0.75; light purple: 0.75≥ρ>0.5; gray: 0.5≥ρ>0; red-brown: ρ<0. **(C)** Dot plot of SOMAscan and PEA data (n=50).

**Figure 6:**
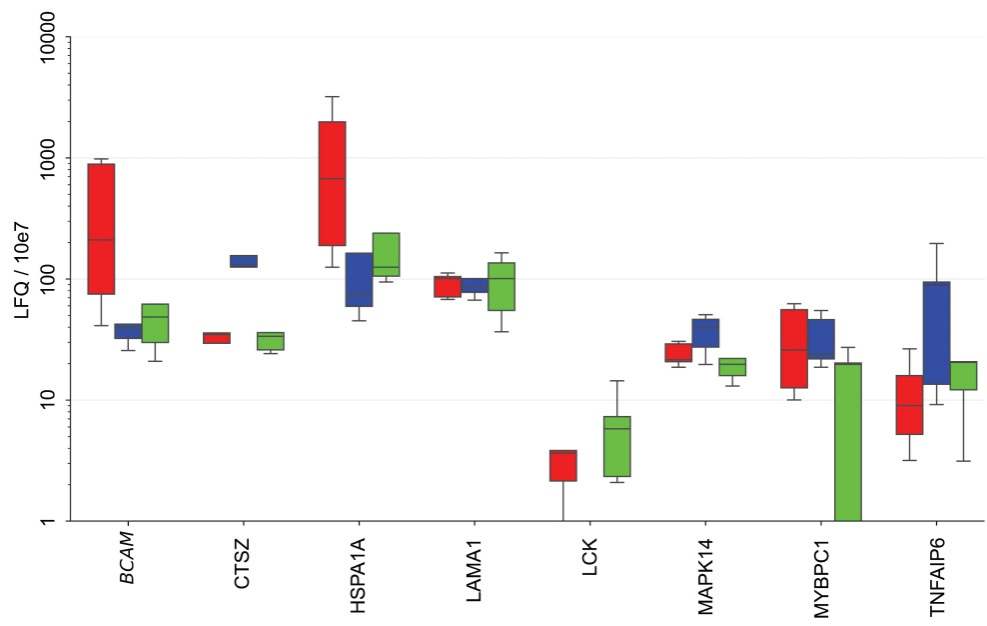
** Secretion of RFS-associated proteins by tumor cells, TAMs and TATs from HGSC patients.** Conditioned medium from primary cells cultured for 5 hrs in protein-free medium was analyzed by LC-MS/MS (n=5 for each cell type). Boxplots show medians (horizontal line in boxes), upper and lower quartiles (box) and range (whiskers). The analysis was carried out with the top 20 RFS-associated proteins. CA4, DKK1, L1A, IL36A and PRSS22 are not shown because they were not detectable in any of the secretomes. Tu: tumor cells, TAM: tumor associated macrophage, TAT tumor associated T-cells.

**Figure 7:**
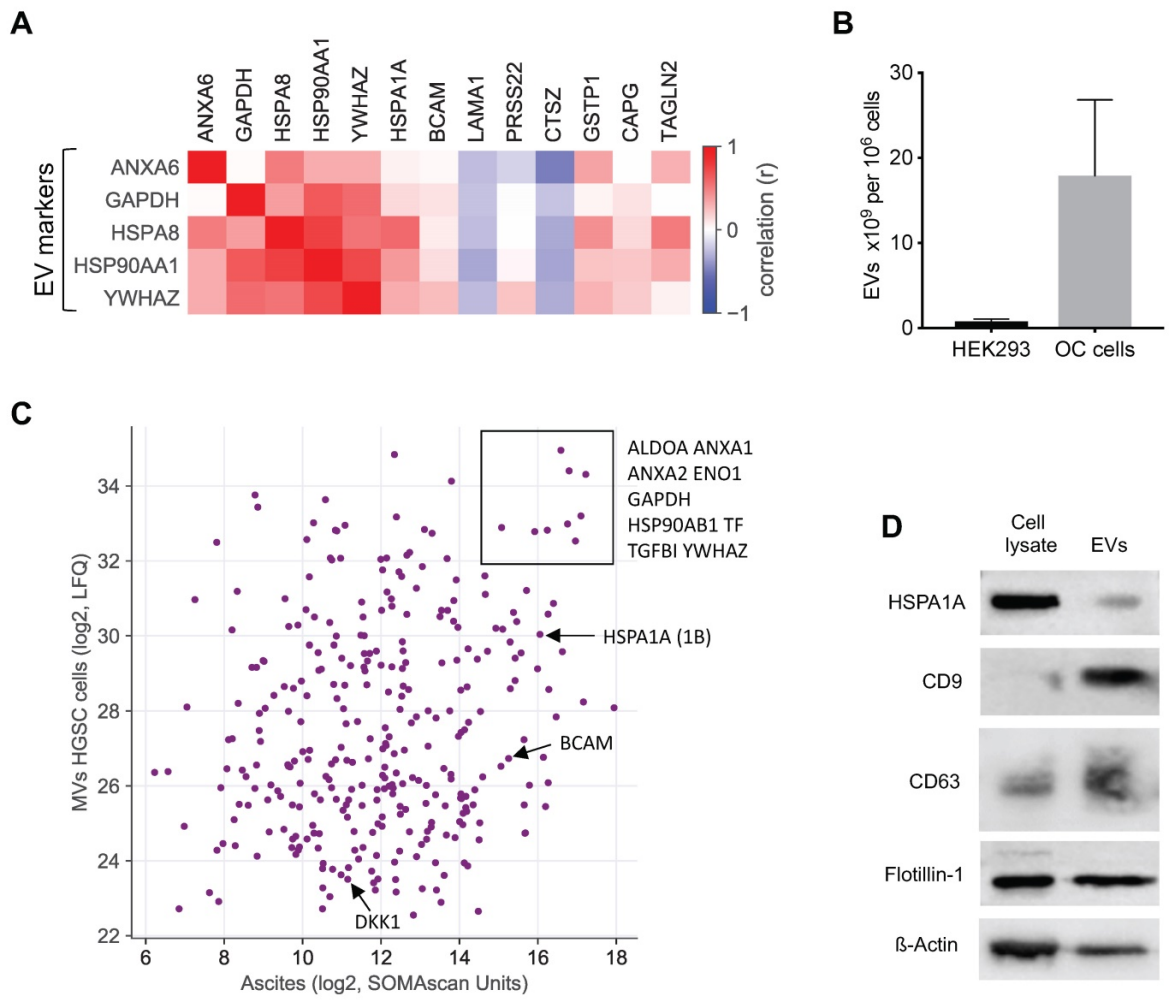
** EVs as the putative origin of RFS-associated proteins. (A)** Heatmap depicting the correlation (Spearman ρ on SOMAscan ascites samples) of EV markers with highRFS-associated SOMAscan protein signals (median concentrations >10.000 SOMAscan units). **(B)** EV numbers in the supernatant of HEK293 cells (n=4) and HGSC cells (n=6) determined by Nanoparticle Tracking Analysis. **(C)** Proteins present in EVs from HGSC tumor cells and in HGSC ascites. Median LFQ values determined by MS are plotted against median SOMAscan units. Rectangle: proteins with the highest concentration in both EVs and ascites. Arrows indicate the data points for BCAM, DKK1 and HSPA1A. **(D)** Detection of HSPA1A in EVs by Western blotting. CD9, CD63 and flotillin were included as known constituents of EVs and ß-actin as a loading control.

**Figure 8:**
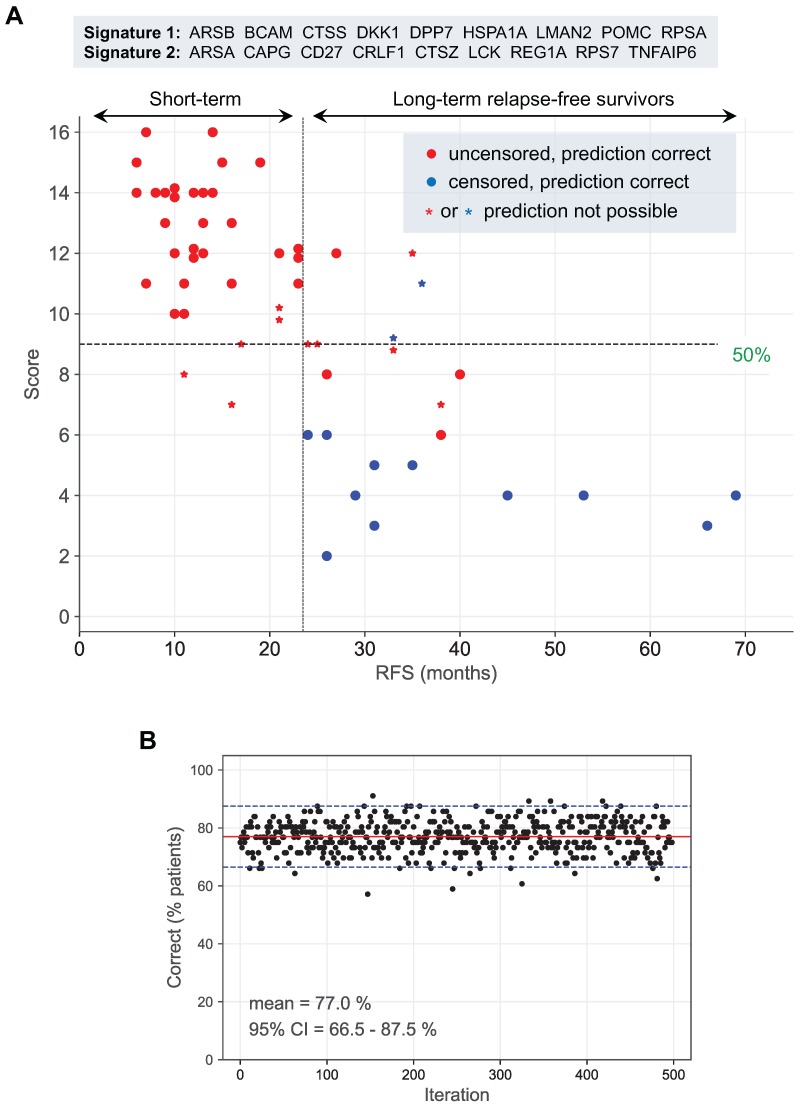
** Prediction of long-term and short-term relapse-free survivors by a combination of two signatures. (A)** Patients for which both signature 1 and 2 (ID = 10 in Table S7) were inconsistent (see main text and Materials and Methods for details) were considered as "prediction not predictable". For consistent instances, predictions were considered either "short RFS" for scores (added signature 1 and 2 scores) above 50% of the maximally possible score, or “long RFS” for scores below 50% of the maximally possible score (dashed horizontal line). The maximally possible score is the added length of both signatures. Short-term survivors were defined patients with relapsed cancer within 24 months after first-line surgery (uncensored RFS <24 months). Long-term survivors are patients remaining relapse-free for at least 24 months (censored or uncensored RFS ≥24 months). The 24-months threshold is indicated by a dashed vertical line. **(B)** Bootstrapping analysis testing the performance of the same signatures as in panel A with 500 resampled sets of patients. Red line: median; blue lines: 95% CI interval.

One aim of the present study was to assess whether proteins in ascites might be useful for the identification of long-term and short-term relapse-free survivors of HGSC. Since none of the proteins associated with RFS on their own was individually able to distinguish these groups of patients with satisfactory accuracy, we took an unbiased approach to define multi-protein signatures. Even though we were unable to identify a single combination that precisely discriminated these patients, we found signatures that identified either long-term or all short-term relapse-free survivors with 100% sensitivity, albeit with false positives in both cases. Based on this observation we identified combination of the two types of signatures that reliably discriminated both groups of patients (Fig. 5). Many of the protein signals strongly associated with RFS (Table 2) are part of these 9-marker signatures, including BCAM, CTSZ, HSPA1A and LCK discussed above, but also proteins with a much lower logrank p-value, e.g., ARSA, CD27 and POMC. This result emphasizes the potential of combinatorial approaches based on large numbers of markers, since it obviously attenuates the impact of outliers in single-marker associations.

The signatures defined by our work may also provide a basis for the development of prognostic tools and may facilitate the establishment of individualized therapies. Although our findings demonstrate the power of combining protein biomarkers in ascites to predict the clinical course of HGSC patients, further improvements by, for instance, analyzing larger panels of proteins are well possible. To unequivocally identify the best possible signature(s) and to prove their prognostic or predictive value it will also be required to analyze large independent cohorts of patients and perform prospective clinical studies.

## Supplementary Material

Supplementary figures and tables.

## Figures and Tables

**Table 1:** Proteins present in ascites at >5-fold higher levels relative to plasma. The indicated p-values were determined by two-sided unpaired t-test and adjusted for multiple hypothesis testing by Benjamini-Hochberg correction. FC: fold change (ratio ascites / plasma).

Protein	FC	p adjusted	Description
IL6	89.8	1.8E-09	interleukin 6
TIMP1	32.0	1.7E-34	TIMP metallopeptidase inhibitor 1
CCDC80	21.7	9.7E-17	coiled-coil domain containing 80
KLK11	15.3	1.5E-19	kallikrein-related peptidase 11
NBL1	15.1	1.1E-09	neuroblastoma 1, DAN family BMP antagonist
GPC3	12.8	8.9E-10	glypican 3
HSPD1	11.4	2.0E-20	heat shock 60kDa protein 1
RSPO3	11.0	3.9E-16	R-spondin 3
NLGN4X	10.3	2.6E-10	neuroligin 4, X-linked
PFDN5	9.8	2.8E-13	prefoldin subunit 5
VEGF121	9.4	3.0E-10	vascular endothelial growth factor alpha
LTA4H	9.1	8.4E-05	leukotriene A4 hydrolase
ANXA2	9.0	7.4E-25	annexin A2
HSP90AA1	8.7	1.1E-16	heat shock protein 90kDa alpha (cytosolic), class A member 1
GAS1	8.4	1.4E-26	growth arrest-specific 1
XPNPEP1	7.8	1.3E-10	X-prolyl aminopeptidase (aminopeptidase P) 1, soluble
CXCL8	7.5	2.4E-08	interleukin 8
STAT1	6.8	3.8E-09	signal transducer and activator of transcription 1, 91kDa
RPS7	6.7	8.4E-10	ribosomal protein S7
NAMPT	6.6	1.2E-05	nicotinamide phosphoribosyltransferase
PRKACA	6.5	2.5E-09	protein kinase, cAMP-dependent, catalytic, alpha
THBS2	6.5	2.4E-25	thrombospondin 2
PDXK	6.4	1.6E-07	pyridoxal (pyridoxine, vitamin B6) kinase
HSPA1A	6.4	1.6E-15	heat shock 70kDa protein 1A
KLK8	6.2	7.2E-11	kallikrein-related peptidase 8
LAMA1	6.1	8.1E-20	laminin, alpha 1
SPOCK2	6.0	4.4E-05	sparc/osteonectin, cwcv and kazal-like domains proteoglycan 2
PRSS22	6.0	1.1E-05	protease, serine, 22
HNRNPA2B1	6.0	8.5E-11	heterogeneous nuclear ribonucleoprotein A2/B1
KPNB1	5.9	7.2E-14	karyopherin (importin) beta 1
CAPG	5.8	1.7E-08	capping protein (actin filament), gelsolin-like
PRKCI	5.7	2.1E-07	protein kinase C, iota
DYNLRB1	5.7	8.8E-08	dynein, light chain, roadblock-type 1
HSPB1	5.6	3.4E-09	heat shock 27kDa protein 1
SCGF-alpha	5.5	1.5E-12	C-Type Lectin Domain Containing 11A (CLEC11A)
ISG15	5.4	1.6E-08	ISG15 ubiquitin-like modifier
PRKCZ	5.4	5.1E-09	protein kinase C, zeta
ANGPT2	5.3	1.1E-16	angiopoietin 2
Thrombin	5.3	1.2E-07	Thrombin
EIF4G2	5.3	3.4E-09	eukaryotic translation initiation factor 4 gamma, 2
EIF4H	5.2	1.1E-10	eukaryotic translation initiation factor 4H
TNFSF8	5.1	2.2E-20	tumor necrosis factor (ligand) superfamily, member 8
CXCL13	5.0	8.2E-09	chemokine (C-X-C motif) ligand 13
HNRNPAB	5.0	2.5E-05	heterogeneous nuclear ribonucleoprotein A/B
TPI1	5.0	4.2E-10	triosephosphate isomerase 1

**Table 2:** Top 50 proteins associated with RFS of HGSC patients. q: best-fit quantile for dichotomization of samples; p: logrank p-value; HR: hazard ratio.

Protein	q	p	HR
HSPA1A	0.6	7.8E-06	3.95
BCAM	0.7	2.6E-05	3.67
DKK1	0.5	2.6E-04	3.23
TNFAIP6	0.4	3.2E-04	3.34
LCK	0.6	4.3E-04	0.29
MYBPC1	0.4	4.9E-04	0.34
LAMA1	0.5	5.3E-04	3.10
PRSS22	0.4	6.2E-04	3.04
CTSZ	0.5	6.2E-04	2.89
MAPK14	0.4	1.1E-03	0.38
MMP16	0.5	1.2E-03	0.36
IL1A	0.4	1.3E-03	0.36
GSTP1	0.7	1.3E-03	2.84
RET	0.7	1.4E-03	2.71
CAPG	0.3	1.5E-03	3.72
IL36A	0.4	1.5E-03	0.38
CHRDL1	0.3	1.6E-03	0.38
CA4	0.5	1.8E-03	2.81
TAGLN2	0.7	2.1E-03	2.77
STK17B	0.5	2.3E-03	0.38
CCL13	0.5	2.4E-03	0.37
CAMK2D	0.6	2.4E-03	0.34
CAMK2B	0.6	2.4E-03	0.34
SEMA5A	0.3	2.4E-03	3.28
PLXNB2	0.3	2.4E-03	3.10
CLIC1	0.6	2.5E-03	0.35
STAT6	0.5	2.5E-03	0.39
NOV	0.3	2.5E-03	3.27
CSK	0.6	2.6E-03	0.35
Fibrinogen	0.6	2.7E-03	2.42
FGG	0.3	2.9E-03	2.99
CNTN2	0.5	3.0E-03	2.58
CAT	0.4	3.2E-03	0.40
SERPINE2	0.4	3.4E-03	0.40
IL18R1	0.7	3.5E-03	2.58
RAC3	0.3	3.6E-03	0.39
NAGK	0.6	3.6E-03	0.36
KLK3	0.5	3.6E-03	0.40
FN1.4	0.4	3.6E-03	2.66
PLCG1	0.6	3.7E-03	0.37
CLEC1B	0.6	4.1E-03	2.35
STAT3	0.6	4.4E-03	0.38
FAS	0.4	4.4E-03	2.60
CAMK2A	0.6	4.4E-03	0.38
FN1.3	0.3	4.6E-03	2.80
DSG1	0.3	4.6E-03	3.26
CA13	0.7	4.7E-03	0.32
WNK3	0.6	4.8E-03	0.38
REG1A	0.3	4.8E-03	0.42
CPB2	0.5	5.0E-03	0.42

## References

[1] Colombo N, Peiretti M, Parma G, Lapresa M, Mancari R, Carinelli S (2010). Newly diagnosed and relapsed epithelial ovarian carcinoma: ESMO Clinical Practice Guidelines for diagnosis, treatment and follow-up. Ann Oncol.

[2] Worzfeld T, Pogge von Strandmann E, Huber M, Adhikary T, Wagner U, Reinartz S (2017). The Unique Molecular and Cellular Microenvironment of Ovarian Cancer. Front Oncol.

[3] Vaughan S, Coward JI, Bast RC Jr, Berchuck A, Berek JS, Brenton JD (2011). Rethinking ovarian cancer: recommendations for improving outcomes. Nat Rev Cancer.

[4] Ahmed N, Stenvers KL (2013). Getting to Know Ovarian Cancer Ascites: Opportunities for Targeted Therapy-Based Translational Research. Front Oncol.

[5] Kipps E, Tan DS, Kaye SB (2013). Meeting the challenge of ascites in ovarian cancer: new avenues for therapy and research. Nat Rev Cancer.

[6] Pogge von Strandmann E, Reinartz S, Wager U, Müller R (2017). Tumor-Host Cell Interactions in Ovarian Cancer: Pathways to Therapy Failure. Trends Cancer.

[7] Finkernagel F, Reinartz S, Lieber S, Adhikary T, Wortmann A, Hoffmann N (2016). The transcriptional signature of human ovarian carcinoma macrophages is associated with extracellular matrix reorganization. Oncotarget.

[8] Worzfeld T, Finkernagel F, Reinartz S, Konzer A, Adhikary T, Nist A (2018). Proteotranscriptomics Reveal Signaling Networks in the Ovarian Cancer Microenvironment. Mol Cell Proteomics.

[9] Scambia G, Testa U, Benedetti Panici P, Foti E, Martucci R, Gadducci A (1995). Prognostic significance of interleukin 6 serum levels in patients with ovarian cancer. Br J Cancer.

[10] Lane D, Matte I, Rancourt C, Piche A (2011). Prognostic significance of IL-6 and IL-8 ascites levels in ovarian cancer patients. BMC Cancer.

[11] Lo CW, Chen MW, Hsiao M, Wang S, Chen CA, Hsiao SM (2011). IL-6 trans-signaling in formation and progression of malignant ascites in ovarian cancer. Cancer Res.

[12] Yanaihara N, Anglesio MS, Ochiai K, Hirata Y, Saito M, Nagata C (2012). Cytokine gene expression signature in ovarian clear cell carcinoma. Int J Oncol.

[13] Reinartz S, Schumann T, Finkernagel F, Wortmann A, Jansen JM, Meissner W (2014). Mixed-polarization phenotype of ascites-associated macrophages in human ovarian carcinoma: Correlation of CD163 expression, cytokine levels and early relapse. Int J Cancer.

[14] Isobe A, Sawada K, Kinose Y, Ohyagi-Hara C, Nakatsuka E, Makino H (2015). Interleukin 6 receptor is an independent prognostic factor and a potential therapeutic target of ovarian cancer. PLoS One.

[15] Reinartz S, Finkernagel F, Adhikary T, Rohnalter V, Schumann T, Schober Y (2016). A transcriptome-based global map of signaling pathways in the ovarian cancer microenvironment associated with clinical outcome. Genome Biol.

[16] Shender VO, Pavlyukov MS, Ziganshin RH, Arapidi GP, Kovalchuk SI, Anikanov NA (2014). Proteome-metabolome profiling of ovarian cancer ascites reveals novel components involved in intercellular communication. Mol Cell Proteomics.

[17] Bery A, Leung F, Smith CR, Diamandis EP, Kulasingam V (2014). Deciphering the ovarian cancer ascites fluid peptidome. Clin Proteomics.

[18] Elschenbroich S, Ignatchenko V, Clarke B, Kalloger SE, Boutros PC, Gramolini AO (2011). In-depth proteomics of ovarian cancer ascites: combining shotgun proteomics and selected reaction monitoring mass spectrometry. J Proteome Res.

[19] Kuk C, Kulasingam V, Gunawardana CG, Smith CR, Batruch I, Diamandis EP (2009). Mining the ovarian cancer ascites proteome for potential ovarian cancer biomarkers. Mol Cell Proteomics.

[20] Gortzak-Uzan L, Ignatchenko A, Evangelou AI, Agochiya M, Brown KA, St Onge P (2008). A proteome resource of ovarian cancer ascites: integrated proteomic and bioinformatic analyses to identify putative biomarkers. J Proteome Res.

[21] Cress RD, Chen YS, Morris CR, Petersen M, Leiserowitz GS (2015). Characteristics of Long-Term Survivors of Epithelial Ovarian Cancer. Obstet Gynecol.

[22] Dao F, Schlappe BA, Tseng J, Lester J, Nick AM, Lutgendorf SK (2016). Characteristics of 10-year survivors of high-grade serous ovarian carcinoma. Gynecol Oncol.

[23] Tewari D, Java JJ, Salani R, Armstrong DK, Markman M, Herzog T (2015). Long-term survival advantage and prognostic factors associated with intraperitoneal chemotherapy treatment in advanced ovarian cancer: a gynecologic oncology group study. J Clin Oncol.

[24] Suidan RS, Ramirez PT, Sarasohn DM, Teitcher JB, Mironov S, Iyer RB (2014). A multicenter prospective trial evaluating the ability of preoperative computed tomography scan and serum CA-125 to predict suboptimal cytoreduction at primary debulking surgery for advanced ovarian, fallopian tube, and peritoneal cancer. Gynecologic oncology.

[25] Barlin JN, Long KC, Tanner EJ, Gardner GJ, Leitao MM Jr, Levine DA (2013). Optimal (</=1 cm) but visible residual disease: is extensive debulking warranted?. Gynecologic oncology.

[26] Waldron L, Haibe-Kains B, Culhane AC, Riester M, Ding J, Wang XV (2014). Comparative meta-analysis of prognostic gene signatures for late-stage ovarian cancer.

[27] Barlin JN, Jelinic P, Olvera N, Bogomolniy F, Bisogna M, Dao F (2013). Validated gene targets associated with curatively treated advanced serous ovarian carcinoma. Gynecologic oncology.

[28] Verhaak RG, Tamayo P, Yang JY, Hubbard D, Zhang H, Creighton CJ (2013). Prognostically relevant gene signatures of high-grade serous ovarian carcinoma. J Clin Invest.

[29] Lollo B, Steele F, Gold L (2014). Beyond antibodies: new affinity reagents to unlock the proteome. Proteomics.

[30] Assarsson E, Lundberg M, Holmquist G, Bjorkesten J, Thorsen SB, Ekman D (2014). Homogenous 96-plex PEA immunoassay exhibiting high sensitivity, specificity, and excellent scalability. PLoS One.

[31] Ince TA, Sousa AD, Jones MA, Harrell JC, Agoston ES, Krohn M (2015). Characterization of twenty-five ovarian tumour cell lines that phenocopy primary tumours. Nat Commun.

[32] Bachurski D, Schuldner M, Nguyen PH, Malz A, Reiners KS, Grenzi PC (2019). Extracellular vesicle measurements with nanoparticle tracking analysis - An accuracy and repeatability comparison between NanoSight NS300 and ZetaView. J Extracell Vesicles.

[33] Gold L, Ayers D, Bertino J, Bock C, Bock A, Brody EN (2010). Aptamer-based multiplexed proteomic technology for biomarker discovery. PLoS One.

[34] Kraemer S, Vaught JD, Bock C, Gold L, Katilius E, Keeney TR (2011). From SOMAmer-based biomarker discovery to diagnostic and clinical applications: a SOMAmer-based, streamlined multiplex proteomic assay. PLoS One.

[35] Welton JL, Brennan P, Gurney M, Webber JP, Spary LK, Carton DG (2016). Proteomics analysis of vesicles isolated from plasma and urine of prostate cancer patients using a multiplex, aptamer-based protein array. J Extracell Vesicles.

[36] Suhre K, Arnold M, Bhagwat AM, Cotton RJ, Engelke R, Raffler J (2017). Connecting genetic risk to disease end points through the human blood plasma proteome. Nat Commun.

[37] Billing AM, Ben Hamidane H, Bhagwat AM, Cotton RJ, Dib SS, Kumar P (2017). Complementarity of SOMAscan to LC-MS/MS and RNA-seq for quantitative profiling of human embryonic and mesenchymal stem cells. J Proteomics.

[38] Sun BB, Maranville JC, Peters JE, Stacey D, Staley JR, Blackshaw J (2018). Genomic atlas of the human plasma proteome. Nature.

[39] Coward J, Kulbe H, Chakravarty P, Leader D, Vassileva V, Leinster DA (2011). Interleukin-6 as a therapeutic target in human ovarian cancer. Clin Cancer Res.

[40] Rauvala M, Puistola U, Turpeenniemi-Hujanen T (2005). Gelatinases and their tissue inhibitors in ovarian tumors; TIMP-1 is a predictive as well as a prognostic factor. Gynecol Oncol.

[41] Shigemasa K, Gu L, Tanimoto H, O'Brien TJ, Ohama K (2004). Human kallikrein gene 11 (KLK11) mRNA overexpression is associated with poor prognosis in patients with epithelial ovarian cancer. Clin Cancer Res.

[42] Geng X, Liu Y, Diersch S, Kotzsch M, Grill S, Weichert W (2017). Clinical relevance of kallikrein-related peptidase 9, 10, 11, and 15 mRNA expression in advanced high-grade serous ovarian cancer. PLoS One.

[43] Jang K, Kim M, Gilbert CA, Simpkins F, Ince TA, Slingerland JM (2017). VEGFA activates an epigenetic pathway upregulating ovarian cancer-initiating cells. EMBO Mol Med.

[44] Komatsu H, Oishi T, Itamochi H, Shimada M, Sato S, Chikumi J (2017). Serum Vascular Endothelial Growth Factor-A as a Prognostic Biomarker for Epithelial Ovarian Cancer. Int J Gynecol Cancer.

[45] Song G, Cai QF, Mao YB, Ming YL, Bao SD, Ouyang GL (2008). Osteopontin promotes ovarian cancer progression and cell survival and increases HIF-1alpha expression through the PI3-K/Akt pathway. Cancer Sci.

[46] Mi H, Dong Q, Muruganujan A, Gaudet P, Lewis S, Thomas PD (2010). PANTHER version 7: improved phylogenetic trees, orthologs and collaboration with the Gene Ontology Consortium. Nucleic Acids Res.

[47] Dalal V, Kumar R, Kumar S, Sharma A, Kumar L, Sharma JB (2018). Biomarker potential of IL-6 and VEGF-A in ascitic fluid of epithelial ovarian cancer patients. Clin Chim Acta.

[48] Reinartz S, Lieber S, Pesek J, Brandt DT, Asafova A, Finkernagel F (2018). Cell-type-selective pathways and clinical associations of lysophosphatidic acid biosynthesis and signaling in the ovarian cancer microenvironment.

[49] Mathivanan S, Lim JW, Tauro BJ, Ji H, Moritz RL, Simpson RJ (2010). Proteomics analysis of A33 immunoaffinity-purified exosomes released from the human colon tumor cell line LIM1215 reveals a tissue-specific protein signature. Molecular & cellular proteomics: MCP.

[50] Yakimchuk K (2015). Exosomes: isolation and characterization methods and specific markers.

[51] Yanez-Mo M, Siljander PR, Andreu Z, Zavec AB, Borras FE, Buzas EI (2015). Biological properties of extracellular vesicles and their physiological functions. J Extracell Vesicles.

[52] Welton JL, Khanna S, Giles PJ, Brennan P, Brewis IA, Staffurth J (2010). Proteomics analysis of bladder cancer exosomes. Mol Cell Proteomics.

[53] Webber J, Stone TC, Katilius E, Smith BC, Gordon B, Mason MD (2014). Proteomics analysis of cancer exosomes using a novel modified aptamer-based array (SOMAscan) platform. Mol Cell Proteomics.

[54] Herath NI, Spanevello MD, Sabesan S, Newton T, Cummings M, Duffy S (2006). Over-expression of Eph and ephrin genes in advanced ovarian cancer: ephrin gene expression correlates with shortened survival. BMC Cancer.

[55] Rudlowski C, Pickart AK, Fuhljahn C, Friepoertner T, Schlehe B, Biesterfeld S (2006). Prognostic significance of vascular endothelial growth factor expression in ovarian cancer patients: a long-term follow-up. Int J Gynecol Cancer.

[56] Aune G, Lian AM, Tingulstad S, Torp SH, Forsmo S, Reseland JE (2011). Increased circulating hepatocyte growth factor (HGF): a marker of epithelial ovarian cancer and an indicator of poor prognosis. Gynecol Oncol.

[57] Gooden MJ, Wiersma VR, Boerma A, Leffers N, Boezen HM, ten Hoor KA (2014). Elevated serum CXCL16 is an independent predictor of poor survival in ovarian cancer and may reflect pro-metastatic ADAM protease activity. Br J Cancer.

[58] Moran-Jones K, Gloss BS, Murali R, Chang DK, Colvin EK, Jones MD (2015). Connective tissue growth factor as a novel therapeutic target in high grade serous ovarian cancer. Oncotarget.

[59] Wang Q, Tang Y, Yu H, Yin Q, Li M, Shi L (2016). CCL18 from tumor-cells promotes epithelial ovarian cancer metastasis via mTOR signaling pathway. Mol Carcinog.

[60] Shizhuo W, Tao J, Shulan Z, Bing Z (2009). The expression and significance of Dickkopf-1 in epithelial ovarian carcinoma. Int J Biol Markers.

[61] Kagey MH, He X (2017). Rationale for targeting the Wnt signalling modulator Dickkopf-1 for oncology. Br J Pharmacol.

[62] Radons J (2016). The human HSP70 family of chaperones: where do we stand?. Cell Stress Chaperones.

[63] Lancaster GI, Febbraio MA (2005). Exosome-dependent trafficking of HSP70: a novel secretory pathway for cellular stress proteins. J Biol Chem.

[64] Yoshioka Y, Konishi Y, Kosaka N, Katsuda T, Kato T, Ochiya T (2013). Comparative marker analysis of extracellular vesicles in different human cancer types.

[65] Murshid A, Theriault J, Gong J, Calderwood SK (2011). Investigating receptors for extracellular heat shock proteins. Methods Mol Biol.

[66] Theriault JR, Mambula SS, Sawamura T, Stevenson MA, Calderwood SK (2005). Extracellular HSP70 binding to surface receptors present on antigen presenting cells and endothelial/epithelial cells. FEBS Lett.

[67] Shevtsov M, Huile G, Multhoff G (2018). Membrane heat shock protein 70: a theranostic target for cancer therapy.

[68] Kikkawa Y, Miner JH (2005). Review: Lutheran/B-CAM: a laminin receptor on red blood cells and in various tissues. Connect Tissue Res.

[69] Miura Y, Matsui S, Miyata N, Harada K, Kikkawa Y, Ohmuraya M (2018). Differential expression of Lutheran/BCAM regulates biliary tissue remodeling in ductular reaction during liver regeneration.

[70] Kikkawa Y, Ogawa T, Sudo R, Yamada Y, Katagiri F, Hozumi K (2013). The lutheran/basal cell adhesion molecule promotes tumor cell migration by modulating integrin-mediated cell attachment to laminin-511 protein. J Biol Chem.

[71] Collec E, Lecomte MC, El Nemer W, Colin Y, Le Van Kim C (2011). Novel role for the Lu/BCAM-spectrin interaction in actin cytoskeleton reorganization. Biochem J.

[72] Bartolini A, Cardaci S, Lamba S, Oddo D, Marchio C, Cassoni P (2016). BCAM and LAMA5 Mediate the Recognition between Tumor Cells and the Endothelium in the Metastatic Spreading of KRAS-Mutant Colorectal Cancer. Clin Cancer Res.

[73] Olson OC, Joyce JA (2015). Cysteine cathepsin proteases: regulators of cancer progression and therapeutic response. Nat Rev Cancer.

[74] Santamaria I, Velasco G, Pendas AM, Fueyo A, Lopez-Otin C (1998). Cathepsin Z, a novel human cysteine proteinase with a short propeptide domain and a unique chromosomal location. J Biol Chem.

[75] Akkari L, Gocheva V, Kester JC, Hunter KE, Quick ML, Sevenich L (2014). Distinct functions of macrophage-derived and cancer cell-derived cathepsin Z combine to promote tumor malignancy via interactions with the extracellular matrix. Genes Dev.

[76] Sevenich L, Schurigt U, Sachse K, Gajda M, Werner F, Muller S (2010). Synergistic antitumor effects of combined cathepsin B and cathepsin Z deficiencies on breast cancer progression and metastasis in mice. Proc Natl Acad Sci U S A.

[77] Wang J, Chen L, Li Y, Guan XY (2011). Overexpression of cathepsin Z contributes to tumor metastasis by inducing epithelial-mesenchymal transition in hepatocellular carcinoma. PLoS One.

[78] Losch A, Schindl M, Kohlberger P, Lahodny J, Breitenecker G, Horvat R (2004). Cathepsin D in ovarian cancer: prognostic value and correlation with p53 expression and microvessel density. Gynecol Oncol.

[79] Baekelandt M, Holm R, Trope CG, Nesland JM, Kristensen GB (1999). The significance of metastasis-related factors cathepsin-D and nm23 in advanced ovarian cancer. Ann Oncol.

[80] Scambia G, Panici PB, Ferrandina G, Salerno G, D'Agostino G, Distefano M (1994). Clinical significance of cathepsin D in primary ovarian cancer. Eur J Cancer.

[81] Bulavin DV, Fornace AJ Jr (2004). p38 MAP kinase's emerging role as a tumor suppressor. Adv Cancer Res.

[82] Sanjo H, Kawai T, Akira S (1998). DRAKs, novel serine/threonine kinases related to death-associated protein kinase that trigger apoptosis. J Biol Chem.

